# Age-period-cohort analysis for trends in body mass index in Ireland

**DOI:** 10.1186/1471-2458-13-889

**Published:** 2013-09-25

**Authors:** Tao Jiang, Mark S Gilthorpe, Frances Shiely, Janas M Harrington, Ivan J Perry, Cecily C Kelleher, Yu-Kang Tu

**Affiliations:** 1 Division of Epidemiology & Biostatistics, School of Medicine, University of Leeds, Room 8.49, Level 8, Worsley Building, Leeds LS2 9JT, UK; 2 University College Cork, Cork, Republic of Ireland; 3 School of Public Health, Physiotherapy and Population Science, University College Dublin, Dublin, Republic of Ireland; 4 Institute of Epidemiology & Preventive Medicine, College of Public Health, National Taiwan University, Taipei, Taiwan

**Keywords:** Obesity, Age-period-cohort, Partial least squares

## Abstract

**Background:**

Obesity is a growing problem worldwide and can often result in a variety of negative health outcomes. In this study we aim to apply partial least squares (PLS) methodology to estimate the separate effects of age, period and cohort on the trends in obesity as measured by body mass index (BMI).

**Methods:**

Using PLS we will obtain gender specific linear effects of age, period and cohort on obesity. We also explore and model nonlinear relationships of BMI with age, period and cohort. We analysed the results from 7,796 men and 10,220 women collected through the SLAN (Surveys of Lifestyle, attitudes and Nutrition) in Ireland in the years 1998, 2002 and 2007.

**Results:**

PLS analysis revealed a positive period effect over the years. Additionally, men born later tended to have lower BMI (−0.026 kg·m^-2^ yr^-1^, 95% CI: -0.030 to −0.024) and older men had in general higher BMI (0.029 kg·m^-2^ yr^-1^, 95% CI: 0.026 to 0.033). Similarly for women, those born later had lower BMI (−0.025 kg·m^-2^ yr^-1^, 95% CI: -0.029 to −0.022) and older women in general had higher BMI (0.029 kg·m^-2^ yr^-1^, 95% CI: 0.025 to 0.033). Nonlinear analyses revealed that BMI has a substantial curvilinear relationship with age, though less so with birth cohort.

**Conclusion:**

We notice a generally positive age and period effect but a slightly negative cohort effect. Knowing this, we have a better understanding of the different risk groups which allows for effective public intervention measures to be designed and targeted for these specific population subgroups.

## Background

Obesity has increased in prevalence worldwide in the last 20 years [1-3] and is associated with a variety of adverse health outcomes [4]. Ireland is no exception in this regard and a recent study has shown a temporal increase in underreporting of overweight and obesity [5]. This has been attributed to an increase in self-reported weight bias which has increased for both sexes and in all age groups. The increased bias is most notable in the obese category [6]. It is valuable to examine these trends in relation to age, period and birth cohort, in conjunction with several evolving environmental factors. Age-period-cohort (APC) analysis is a popular analytic approach in both epidemiological and sociological studies [7,8]. Knowing the separate effects of age, period and cohort allows for a better understanding of, for example, the different risk groups based on age and generation cohort, separately to current and constantly developing environmental factors. The cohort effect can identify individuals who are particularly at high risk of obesity, to allow for effective public intervention measures to be designed and targeted for these specific population subgroups. However, one longstanding problem with APC analysis, as undertaken using standard regression analysis techniques, is perfect collinearity [9], i.e. the intrinsic mathematical relation amongst Age, Period and Cohort: *Period* = *Age* + *cohort*_._

As a direct result of perfect collinearity, the three linear effects are not well defined [10] because, given any two, the third can be exactly computed, and all three cannot be simultaneously estimated within a generalised linear model. For example, if researchers observed a trend in the body mass index of people in Ireland, this could be due to the aging process (age effect), everyone eating more healthily (period effect), or nutritional advice given to mothers (cohort effect). Due to perfect collinearity, we only have two degrees of freedom, despite having three variables. This means that the data matrix is singular, i.e. it is not invertible, hence ordinary linear modelling will not produce unique coefficient estimates [11].

There have been many proposed techniques to deal with this identification problem. The most conceptually simple is to impose a constraint on the parameters in the estimation process [12]. However, while this obtains unique parameter estimates, the choice of constraint greatly affects the estimated coefficient values, and there is no empirical method of differentiating between constraints chosen, since they all yield identical model fit criteria [13]. Parameter interpretation therefore becomes challenging and justification for any specific constraint chosen usually falls to clinical insight, which is not always obvious or available.

Alternatively, although ordinary least squares regression analysis requires that the data matrix be full rank and invertible, this is not a restriction for partial least squares (PLS) regression; hence the problem of perfect collinearity and identification is circumnavigated by PLS [14,15]. The objective of this paper is to use PLS to estimate the separate effects of age, period and birth cohort. Furthermore, we aim to develop the PLS method to accommodate curvilinear effects. The dataset used was collected in Ireland in the years 1998, 2002 and 2007, and includes the age, sex and body mass index (BMI) of each participant. We take BMI as our outcome measure and use PLS regression to obtain estimates for age (age at measurement), period (year of measurement) and cohort (date of birth).

## Methods

### Cohort information

The data were obtained in the Republic of Ireland via three consecutive waves of the Surveys of Lifestyle, Attitudes and Nutrition (SLAN), all of which are now publicly available. Ethical approval for this study was granted by an independent ethical committee established by Department of Health and Children (1998), the Faculty of Public Health Medicine of the Royal College of Physicians (2002) and the Royal College of Surgeons in Ireland (2007). Data were collected in 1998, 2002 and 2007 through standardised protocols. The first two SLAN surveys employed a postal self-administered methodology; this seen as a cost effective strategy. However, due to declining response rates, an interview administered survey was used for the third survey. All three surveys had reasonable response rates (62%, 53% and 62% respectively) and are thought to be representative of the population at the three time points. The methodology for all three surveys has been described previously [16-18]. All three samples were generated through random cluster methodologies at district electoral division level. In 1998 and 2002 the An Post database based on the electoral register was used for sample selection yielding response rates of 62% and 53% respectively. SLAN 2007 consisted of a probabilistic sample in three stages: geographic area, household and ‘next birthday’ participant selection within households [19,20]. The sample frame was the *Geodirectory*, a listing of all residential address in Ireland compiled by the postal service. Face-to-face interviews were conducted with adults aged 18 years and over, interviewed at home (response rate of 62%), randomly selected using a method known as RANSAM [21]. A selection of sampling points based on aggregates in towns was completed, providing a sample of private residential addresses, from which the potential participants within the household were selected at random. We do not believe that gender affected the response rates. In all three surveys, in addition to age and sex, participants were asked to self-report their weight without clothes and their height without shoes, from which participants’ BMI could be calculated. The year of birth was calculated as the year of measurement (1999, 2002 or 2007) minus the age of the participant at that time.

Participants with missing values in sex, age or BMI are excluded from the analyses. Those over the age of 75 were excluded due to sparse data. Data were excluded where the height was less than 1.5 m and greater than 2.0 m and where weight was less than 40 kg and greater than 150 kg, to capture likely data report errors, or data entry errors, that might otherwise skew the results Of the original 22,895 participants, we performed our analysis on 18,016 participants: 7,796 men and 10,220 women.

### Partial least squares

PLS extracts weighted components **t** of the explanatory variables, maximising the covariance between the response variable (in our case BMI) and **t**[22]. Once we obtain the estimated coefficients for the PLS components, the coefficients for the original variables are recovered via algebraic manipulation given the weights used (see Additional file 1). The extracted components are ordered corresponding to the amount of covariance they explain, i.e. the first component explains more covariance than the second, which in turn explains more covariance than the third [11]. Consequently, when large numbers of predictor variables are used, only the first few PLS components are required to explain most of the covariance with the outcome. The algorithm penalises variables that have smaller variances, so if there are large differences in the variances across the variables, it is important to scale the variables before applying PLS.

Should the maximum number of components be chosen, PLS gives an identical output to principal components analysis. However, since PLS components are ranked in order of the covariance with the outcome, the first few components account for of the covariance between the covariates and the outcome; hence it is often justified, in the interest of parsimony, to consider only the first few components. Since PLS aims to maximise the covariance between the component and the outcome variable, it is perfectly reasonable to use the percentage of explained variation in the outcome variable as a gauge for the number of components to be selected. We select the number of components based on small changes in R^2^ resulting from the inclusion of an additional component (see Additional file 1 for further details).

### Data analysis

We began with linear PLS analysis for BMI by including age, year of examination and year of birth as covariates. We performed separate analyses for men and women to allow for sex differences. Since PLS penalises against variables with comparatively smaller variances, all predictor variables were scaled prior to running PLS, though the results are rescaled when presented for ease of interpretation. We then created dummy variables for all the predictor variables (one for each year) to explore potential curvilinear effects. No dummy variable was created for age 18, period 1998 and cohort 1923 for reasons of identifiability; no other constraints were placed on the dummy variables, since this is not required for PLS. Having obtained coefficients for the dummy variables, *loess* curves were fitted to identify curvilinear effects [23-25]. The resulting fitted curves were overlaid on the scatter plot of the parameter estimates to allow for visual comparisons. Data manipulation was performed using Microsoft Excel and PLS regression was undertaken using the software TANAGRA (version 1.4.40, http://eric.univ-lyon2.fr/~ricco/tanagra/en/tanagra.html). Results were exported to the statistical software *R* (version 2.13.1, http://www.r-project.org/index.html) for plotting and fitting *loess* curves.

## Results

Table 1 shows the number of individuals in each age group from each of the three years. We see that none of the age groups have particularly low numbers but the numbers do start to drop off at the oldest group. This was the reason we decided to pose a limit on age since the older age groups will have even fewer individuals if we included them.

**Table 1:** Number of subjects of each age group at each point in time by gender

	**Males**			**Females**		
	**1998**	**2002**	**2007**	**1998**	**2002**	**2007**
18-30	741	378	739	891	616	976
31-40	567	411	731	778	795	1108
41-50	526	500	676	505	729	934
51-60	317	291	554	274	342	744
61-75	347	317	701	383	380	765

Table 2 shows the results from the linear PLS regression for men and women separately. The 95% confidence intervals (CI) reveal that all coefficients were statistically significant at the 5% level. It is notable that there does not seem to be substantial difference between men and women. Weak positive associations were observed for Age and Period with BMI, though a negative association was observed for Cohort. The change in R^2^ for the addition of a second component was only 0.17% in men and 0.18% in women, suggesting that 1-component models would be sufficient and parsimonious.

**Table 2:** **Output from linear PLS analysis for men and women with one and two components with scaled variables for Age, Period and Cohort**^**1**^

**Variables**	**Males**				**Females**			
	**1-Component**		**2-Component**		**1-Component**		**2-Component**	
	**Coefficient**	**95% CI**	**Coefficient**	**95% CI**	**Coefficient**	**95% CI**	**Coefficient**	**95% CI**
Age	0.029	(0.026, 0.033)	0.030	(0.027, 0.034)	0.029	(0.025, 0.033)	0.0293	(0.025, 0.033)
Period	0.039	(0.026, 0.051)	0.079	(0.055, 0.098)	0.055	(0.039, 0.070)	0.1023	(0.078, 0.126)
Cohort	−0.026	(−0.030, -0.024)	−0.024	(−0.028, -0.022)	−0.025	(−0.029, -0.022)	−0.0224	(−0.026, -0.019)
R^2^	4.91%		5.08%		3.36%		3.54%	

1 Explicitly, the components are w1 = 0.029*Age + 0.039*Period-0.026*Cohort and w2 = 0.001*Age + 0.04*Period + 0.002*Cohort for men and w1 = 0.029*Age + 0.055*Period-0.025*Cohort and w2 = 0.0003*Age + 0.0473*Period + 0.002*Cohort for women.

Men who were born later in our dataset had lower BMI than those who were born earlier (−0.026 units/yr, 95% CI: -0.030 to −0.024). Men who were older at the time of examination had in general higher BMI than younger men (0.029 units/yr, 95% CI: 0.026 to 0.033). Similarly for women, those born later had lower BMI (−0.025 units/yr, 95% CI: -0.029 to −0.022) and older women had, in general, higher BMI (0.0293 units/yr, 95% CI: 0.025 to 0.033). It should be noted that while the cohort effect is slightly negative, it is swamped by the very positive period effect.

**Figure 1:**
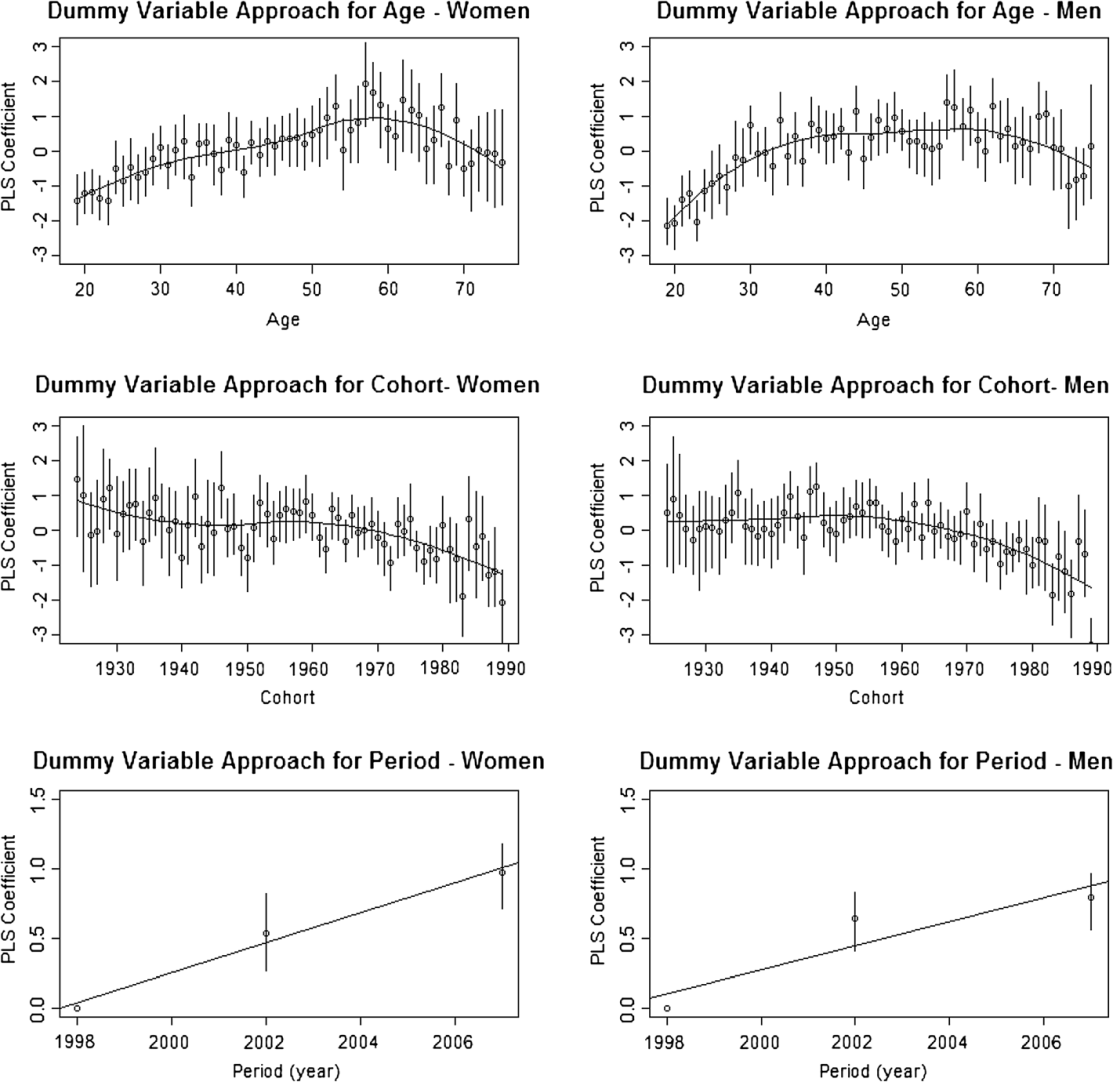
**PLS regression coefficient plot and trend curves for age, period and cohort in men and women.** 4 components were taken based on change in R^2^ for all dummy variable analyses. All parameters were treated as discrete and values rounded to nearest year if they were not integer already. Vertical lines represent 95% confidence intervals.

Performing PLS regression on the dummy variables gave rise to Figure 1. A *loess* curve was fitted to the data points in order to highlight the important features. It can be seen that the trend for cohort is generally negative for both men and women, but there is a distinct peak for women in the age effect. The trend is positive up to age 59 for women, before becoming negative for older women. The overall picture is similar for men, though a distinct peak is not observed; a positive trend is observed up to the age of 35, after which the growth slows and plateaus until age 60, at which point the trend finally becomes negative, as it does for women.

Since we removed the 1998 period from our analysis for identifiability, we have effectively set the value at 1998 to be zero for relative comparison (hence no confidence interval). A linear model was fitted since we have only two other data points. The period plots for both men and women show a distinct positive trend. Gradients of the linear model are very similar to those obtained from our earlier linear analysis.

## Discussion

Our results reveal several features relating to the separate effects of age, period and cohort on BMI in this study population. Whilst the trend in period is positive for both men and women, the rate of increase for women is greater than that for men, though the difference is perhaps marginal. One explanation for the positive trend could be dietary pattern changes that result from an increase in prevalence and accessibility of fast foods and takeaways, yielding an increase in total energy intake. A reduction in physical activities could also contribute to the observed trend.

We have observed that the age effect differs slightly between men and women, but the underlying trend remains similar; indeed the models fit very well. It seems that, in general, lower BMI is observed amongst younger people and BMI steadily increases with age, arriving at a peak that is not as pronounced in men as it is in women. It is possible that this is due to levels of exercise and other day-to-day activities in early life that gradually reduces into midlife [26], or it may be down to underlying genetic, metabolic and/or physiological differences between men and women. After the midlife peak age, however, the trend becomes negative with age, suggesting that older people, on average, have lower levels of BMI compared to midlife values. It is likely, however, that a survival effect is operating for the older ages, since obese people are generally at much higher risk of chronic diseases, such as cardiovascular disease [27], diabetes [28], liver disease [29] and even certain types of cancers [30]. The consequence of this increased risk is that many people with very high levels of BMI may die prematurely and those that survive into later life are therefore likely, on average, to have a healthier BMI. Furthermore, the elderly are much more likely to suffer from loss of muscle mass [31-33]. This effect builds up over time so it is less noticeable in younger individuals but cumulates over time to cause a decrease in BMI estimate for the elderly. This would explain the negative trend in BMI past the peak ages. It is also well known that older adults, particularly women, overestimate their self-reported height more than younger women [34-36], thereby inflating the denominator in a way that could also account for the negative BMI trend past their peak age.

Whilst the fitted curve for men suggests different curvilinear cohort effects than for women, the confidence intervals overlap considerably and the overall patterns look similar; it is thus plausible that the deviation from a linear effect for both men and women is nothing more than chance. It has been suggested that the cohort effect is a reflection of environmental pressures in early life [37]. According to the ‘developmental origins of health and disease’ (DOHaD) hypothesis [38], early life exposures may have a profound effect on later adult health. Indeed, it is hypothesised that if the foetus is subject to certain conditions, such as poor nutrition, it is programmed at birth to expect a harsher environment with potential traits such as lower metabolism, for example. Therefore, if the subsequent childhood environment is not that harsh, the individual is more prone to gain weight and experience an increased BMI [39]. The negative trend in birth cohort we observe may be explained by improved maternal nutrition, which arose because of rapid economic growth in Ireland over the last few decades. As the nutritional needs of the unborn are more readily met over time, the baby will become increasingly correctly ‘programmed’ for the childhood and adulthood environment it faces and is less likely to experience unhealthily elevated BMI. Cohorts born after the introduction of the 1947 Health Act in Ireland, which completely reformed health care delivery, appear to have benefited from improved growth and development patterns in childhood during the 1950s and 60s. There was considerable interest in early childhood nutrition in that period, and a major nutrition survey was first undertaken at the time [40]. Surveillance information suggests improvements in food supply, particularly of fresh and frozen foods, such as fruits and vegetables, through the widespread networking of supermarkets.

The positive secular trends in cardiovascular risk factors seen in many western countries have recently reversed for BMI and plasma glucose [3] and this present analysis suggests that, as the childhood obesity epidemic takes effect, benefits to the young and middle aged seen here in Ireland are not likely to be maintained amongst future younger generations. The age effect represents an unchangeable effect of the ageing process, but also the effects that are unique to each age group yet consistent across all people of that age. Therefore, the age effect is an indicator of which age groups are particularly at risk of obesity, allowing for more targeted intervention methods. Since a decrease in exercise with age has been observed, and the association between exercise and obesity is well documented [41], it is possible that an intervention to persuade older people to maintain active lives and exercise more could be effective.

The cohort effect, on the other hand, can be viewed as a short-lived effect, for which there is only a small window for intervention. Clinically however, it is possible to learn from trends in cohorts, which might demonstrate, for instance, that current practices in prenatal advice and accessibility of relevant information are potentially working quite effectively. A randomised controlled trial in pregnancy to reduce the likelihood of delivering a macrosomic baby has shown positive initial effects on maternal dietary patterns [42], and other Irish cohort data strongly suggest that both familial dietary and BMI patterns cluster closely [43-45]; so there is scope for informed effective intervention.

The period effect is a more gradual long-term effect that impacts all people living through the period; hence it is, with all other things being equal, most susceptible to intervention, since any action will immediately have an effect on the existing population. Indeed, Figure 1 shows that for women, the trend is almost linear and the linear model adopted provides a very good fit. For men, the linear model adopted is not as good a fit and potentially a slight curvilinear effect is emerging, but since we have only three data points, fitting any curvilinear effect will result in a perfect fit; it is not possible therefore to explore the curvilinear effects in period. Despite this, the fitted linear trend passes through both confidence intervals and the fit is reasonable. As we are observing a positive association between period and BMI, an intervention is advisable. It would be possible, for instance, to expose the public to even more information on healthy lifestyles and the impact of poor diets, though the success of health promotion campaigns has had mixed results [46,47]. A weakness in the dataset used in this instance, given only three distinct periods, is the impossibility to investigate any potential curvilinear period effects; hence we cannot access if the observed trend is maintained, accelerating or diminishing.

One limitation in our methodology is that the individual point estimates of the curvilinear analysis cannot be directly compared between plots. This is due to the fact that each plot is generated relative to a particular year or age group. Therefore, while it is possible to compare the results within each plot, it is not possible to compare between plots. Contrasting this is the results of our linear analysis. This is because being the slopes of each effect, it is unaffected by any reference year.

Our data set also only has three points, therefore it was impossible to perform curvilinear analysis on the period effect. We were not interested in the impact of various additional variables of lifestyle as part of this study but this can be investigated in future works.

## Conclusion

Obesity and its associated health implications is a growing concern worldwide. It is well known that obesity prevalence is increasing generally, but through the use of PLS we are able to estimate the separate contributions to obesity of age, period and cohort. The use of PLS to undertake age-period-cohort analysis is simple and direct; the implicit constraint imposed arises directly from the intimate relationship between the three variables (Period = Age + Cohort). Consequently, PLS produces more interpretable results than other approaches (see Additional file 1). This allows the partition of previously observed findings, highlighting those aspects of age, period and cohort that contribute separately towards the overall trend; perhaps reflecting the effect of existing health promotion interventions and highlighting potential strategies for future interventions. Using PLS, we were able to identify that men and women born in Ireland during the period 1924–1989 experienced, on average, a steady increase in BMI by year of age, which became more pronounced throughout the study period, mitigated only slightly by improved maternal nutrition of successive cohorts, which would potentially have given rise to better ‘programming’ of children in preparation for an increasingly obesogenic environment experienced in childhood and adulthood.

## Abbreviations

APC: Age-period-cohort; PLS: Partial least squares; BMI: Body mass index; DOHaD: Developmental origins of health and disease; SLAN: Surveys of lifestyle, attitudes and nutrition; CI: Confidence interval.

## Competing interests

The authors declare that they have no competing interests.

## Authors’ contributions

TJ performed the analysis and drafted the paper under the supervision and guidance of MSG and YKT; JH, CCK and FS helped with the interpretation of the results and IJP provided the data set. All authors contributed to the critical revisions of the draft manuscript. All authors read and approved the final manuscript.

## Pre-publication history

The pre-publication history for this paper can be accessed here:

http://www.biomedcentral.com/1471-2458/13/889/prepub

## Supplementary Material

Additional file 1 Introduction to PLS. Click here for file
