# Supplementary material for: Age-period-cohort analysis for trends in body mass index in Ireland
Source: BMC Public Health. 2013 Sep 25;13:889. doi: 10.1186/1471-2458-13-889 (PMC3852547; doi:10.1186/1471-2458-13-889)
Supplement: Additional file 1 — Introduction to PLS. [file 1471-2458-13-889-S1.docx]

## Additional Files

### Introduction to PLS

The fundamental problem with APC analysis is perfect collinearity amongst the variables age, period and cohort. The direct consequence of this is that the data matrix is not of full rank, i.e. it is singular and therefore not invertible. This means that more commonly used regression models such as ordinary least squares regression, does not work. Partial least squares (PLS) regression is not affected by the singularity of the data matrix hence it can be applied to APC analysis.

PLS attempts to extract weighted components **t** of the explanatory variables, maximising the covariance between the response variable and **t**. For example, for the variables Age, Period and Cohort: **t**_i_ = w_i1_Age + w_i2_Period + w_i3_Cohort, where the weights w_ij_ are subject to the constraints that $\sum_{j=1}^{3} w_{ij}^{2}=1$ and the components are mutually orthogonal (i.e. the pair-wise correlations are zero).

This differs from principal components analysis (PCA), which extracts components based on the amount of variance each component explains within the data matrix. Therefore, given a set of explanatory variables, principle components analysis will always extract the same components regardless of the response variable.

The maximum number of PLS (and PCA) components that can be extracted is equal to the rank of the data matrix. In our case, although we have three variables (age, period and cohort), we only have two degrees of freedom; hence our data matrix is rank two. This is a direct result of perfect collinearity amongst the variables. Therefore, only two components can be extracted.

Having obtained the coefficients for the PLS components, it is necessary to recover the coefficients for the original predictor variables. For example, suppose we take the full 2-component model in our analysis of BMI, we would obtain the following:

$$BMI=\beta_{1}\mathbf{t}_{1}+\beta_{2}\mathbf{t}_{2}$$

$$\begin{matrix} =\beta_{1}(w_{11}Age+w_{12}Period+w_{13}Cohort)+\beta_{2}(w_{21}Age+w_{22}Period+w_{23}Cohort) \\ =(\beta_{1}w_{11}+\beta_{2}w_{21})Age+(\beta_{1}w_{12}+\beta_{2}w_{22})Period+(\beta_{1}w_{13}+\beta_{2}w_{23})Cohort \end{matrix}$$

with $\beta_{1}$ and $\beta_{2}$ being the estimated coefficients for the 1^st^ and 2^nd^ PLS components respectively. We can therefore, extract the separate regression coefficients for Age, for instance, to be $\beta_{1}w_{11}+\beta_{2}w_{21}$ via simple algebraic manipulation.

### Further Properties

Since we have three variables but only two degrees of freedom, the two extracted components for both principal components analysis and PLS span the same space which is why the 2-component model for PLS gives the same output as that for principal components analysis. Although the extracted components from both methods are not the same, since they span the same space, the resulting coefficients for the original covariates will be identical.

Given we are trying to estimate three separate coefficients but we only have two degrees of freedom, a constraint is necessary. PCA (and PLS) implicitly applies the constraint that the sum of two coefficient estimates is equal to the third.

**Theorem 1**: In a classic APC model where the only covariates are age, cohort and period, PCA will obtain parameter estimates $\lambda_{1},\lambda_{2}\mathrm{and}\lambda_{3}$ and $\lambda_{3}$ for age, cohort and period respectively such that $\lambda_{1}+{\lambda_{2}=\lambda}_{3}$.

**Proof**: Let the PCA algorithm produce two components $\left( \begin{matrix} x_{1} \\ x_{2} \\ x_{3} \end{matrix} \right)$ and $\left( \begin{matrix} y_{1} \\ y_{2} \\ y_{3} \end{matrix} \right)$ with coefficients $\beta_{1}{and \beta}_{2}$ and $\beta_{2}$ respectively. Then we have $\begin{matrix} \lambda_{1}=\beta_{1}x_{1}+\beta_{2}y_{1} \\ \lambda_{2}=\beta_{1}x_{2}+\beta_{2}y_{2} \\ \lambda_{3}=\beta_{1}x_{3}+\beta_{2}y_{3} \end{matrix}$ hence it is sufficient to prove $x_{1}+{x_{2}=x}_{3}$ and $y_{1}+{y_{2}=y}_{3}$.

Let A be the variance in Age, C be the variance in Cohort and B be the covariance between Age and Cohort, the covariance matrix for our data will be $\left( \begin{matrix} A & B & A+B \\ B & C & B+C \\ A+B & B+C & A+2B+C \end{matrix} \right)$. The components extracted by PCA are the eigenvectors ordered in order of size of their corresponding eigenvalues. Consider arbitrary eigenvector $\left( \begin{matrix} z_{1} \\ z_{2} \\ z_{3} \end{matrix} \right)$ with corresponding eigenvalue $\lambda$

$$\left(\begin{matrix} A & B & A+B \\ B & C & B+C \\ A+B & B+C & A+2B+C \end{matrix}\right)\left(\begin{matrix} z_{1} \\ z_{2} \\ z_{3} \end{matrix}\right)=\lambda\left(\begin{matrix} z_{1} \\ z_{2} \\ z_{3} \end{matrix}\right)$$

Hence $\left\{ \begin{matrix} Az_{1}+Bz_{2}+(A+B)z_{3}=\lambda z_{1} \\ Bz_{1}+Cz_{2}+(B+C)z_{3}=\lambda z_{2} \\ (A+B)z_{1}+(B+C)z_{2}+(A+2B+C)z_{3}=\lambda z_{3} \end{matrix} \right.$

Summing the first two equations will give you the left hand side of the third, hence we have

$$\lambda(z_{1}+z_{2})=\lambda z_{3}$$

Therefore
 $z_{1}+z_{2}=z_{3}$ or $\lambda=0$

Since PCA will not extract the component with the zero eigenvalue, all extracted components have the property that $z_{1}+z_{2}=z_{3}$ hence we have $x_{1}+{x_{2}=x}_{3}$ and $y_{1}+{y_{2}=y}_{3}$. [*QED*]

PLS is similar to PCA, only that it considers the covariance between the covariates and the outcome. As a result, it inherits the same constraint on the coefficients as does PCA. This constraint is directly inherited from the mathematical relationship amongst the variables; hence it is a reasonable constraint to impose.
